# Dysbiosis in the Human Microbiome of Cholangiocarcinoma

**DOI:** 10.3389/fphys.2021.715536

**Published:** 2021-11-12

**Authors:** Benchen Rao, Tong Ren, Xuemei Wang, Haiyu Wang, Yawen Zou, Ying Sun, Shanshuo Liu, Zhigang Ren, Zujiang Yu

**Affiliations:** ^1^Department of Infectious Diseases, The First Affiliated Hospital of Zhengzhou University, Zhengzhou, China; ^2^Gene Hospital of Henan Province, Precision Medicine Center, The First Affiliated Hospital of Zhengzhou University, Zhengzhou, China; ^3^Department of Breast Surgery, Affiliated Cancer Hospital of Zhengzhou University, Zhengzhou, China

**Keywords:** cholangiocarcinoma (CCA), human microbiome, gut microbiome, bile microbiome, microbial dysbiosis

## Abstract

Cholangiocarcinoma (CCA) is the most common malignant tumor of the biliary system with a very poor prognosis. The human microbiome, which is the sum of the genetic information of human microorganisms, plays an important role in regulating the digestion, absorption, immune response, and metabolism of the host. Increasing evidence indicates a close relationship between CCA and the human microbiome. Specific alterations occur in the human microbiome of patients with CCA. Therefore, in this review, we aimed to summarize the recent evidence on dysbiosis in the human microbiome of CCA. Then, we generalized the effect of *Helicobacter pylori* on CCA. Additionally, the potential mechanism of human microbial dysbiosis promoted the progress of CCA, and its precancerous disease was also explored. Furthermore, the possibility of the human microbiome as a diagnostic and therapeutic target of CCA was discussed.

## Introduction

Cholangiocarcinoma (CCA) is the most common malignant tumor of the biliary system and the second most common primary hepatobiliary malignancy, accounting for 15% of hepatobiliary malignancies (Banales et al., 2020; Siegel et al., 2020). According to the anatomical location, CCA is classified into intrahepatic cholangiocarcinoma (iCCA), perihilar cholangiocarcinoma, and distal cholangiocarcinoma (Razumilava and Gores, 2014). There are no obvious clinical symptoms in the early stage of CCA, and approximately 70% of patients are in the late stage at the time of diagnosis (Nathan et al., 2009). In 2017, it was expected to have 40,710 new cases of hepatobiliary malignancies, of which approximately 28,920 will eventually die from hepatobiliary malignancies in the United States (Islami et al., 2017).

Cholestasis and chronic inflammation lead to malignant transformation of bile duct cells, which is a common way for the tumorigenesis and development of CCA. Conjugated bile acid stimulates the production and secretion of growth factors and inhibition of apoptosis. A variety of proto-oncogenes are activated, leading to the destruction of the intracellular pathways controlled by the proto-oncogenes and cell canceration (Papoutsoglou et al., 2019; Jin et al., 2020). The independent risk factors recognized by CCA mainly include primary sclerosing cholangitis (PSC), hepatobiliary parasites, bile duct stones, choledochal cysts, and carcinogens (i.e., asbestos, dioxins, and nitrosamines). The potential risk factors include liver cirrhosis, hepatitis B virus infection, hepatitis C virus infection, diabetes, obesity, chronic consumption of alcohol, smoking, biliary, and disorders of enteric circulation (Razumilava and Gores, 2014; Blechacz, 2017). However, most patients with CCA were not exposed to relevant risk factors before the tumorigenesis. Therefore, the etiology and pathogenesis of CCA are still unknown. Increasing evidence showed that CCA developed through the accumulation of genetic and epigenetic changes, which were affected by the human microbiome (Jusakul et al., 2017; McKenzie et al., 2017; Banales et al., 2020).

The human microbiome, which is the sum of the genetic information of human microorganisms, mainly includes the microbiome in the intestinal tract, bile duct, oral cavity, vagina, nasal cavity, and skin (Turnbaugh et al., 2007; Human Microbiome Project Consortium, 2012). The human intestine is colonized with a large number (about 10^14^) and a complex structure (over 1,000 species of bacteria) of microbial community (about 1.5 kg) (Hayes et al., 2010). In the process of symbiosis and co-evolution of the intestinal flora and the host, the gut microbiome plays an important role in regulating the digestion, absorption, immune response, and metabolism of the host (Backhed et al., 2005). As for the bile microbiome, the alterations of the bile microbiome will lead to the occurrence and progression of a variety of hepatobiliary and pancreatic and intestinal diseases (Liwinski et al., 2020). At the same time, the metabolism of bile acids and the characteristics of the gut microbiome will be affected by it (Molinero et al., 2019; Serra et al., 2021). Studies on oral (Hayes et al., 2018), vagina (Nene et al., 2019), nasal (Mika et al., 2016), and skin microbiome (Chen et al., 2018) have also made great progress.

In this article, we reviewed and focused on the alterations in the human microbiome of patients with CCA and its precancerous diseases and analyzed the potential mechanism of the development and progress of CCA caused by the dysbiosis of the human microbiome. In addition, we evaluated the potential of the human microbiome as a diagnostic biomarker and therapeutic target for CCA.

### The Close Relation Between Gut Microbiome and Hepatobiliary Diseases

**FIGURE 1:**
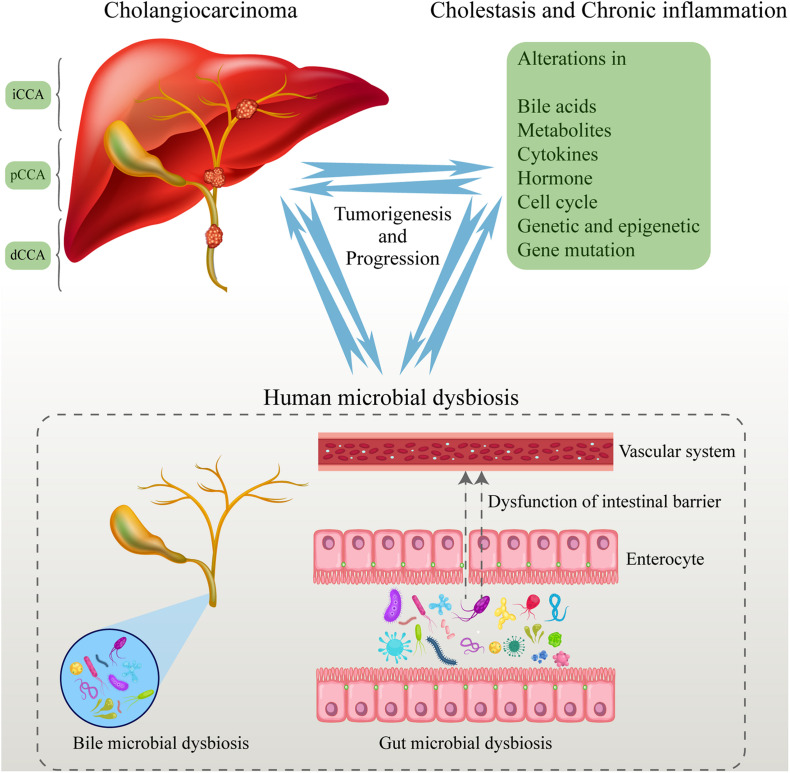
Human microbial dysbiosis promotes the tumorigenesis and progression of CCA. The human microbiome dysbiosis occurred in the process of CCA tumorigenesis. The entry of abnormal microbiome and their metabolites into the blood promotes the progression of CCA. At the same time, CCA aggravates the dysbiosis of the human microbiome by many ways, leading to a vicious circle. CCA, cholangiocarcinoma.

The gut–liver axis and the biliary-enteric circulation are closely related to the function of the hepatobiliary system and gut. The hepatobiliary system has a close relationship with the gut based on the anatomical position and physiological function (Schwabe and Greten, 2020). About 70% of the blood in the hepatobiliary system comes from the portal vein. Nutrients and toxins from the intestine enter the liver through the portal vein, and then they are transported to the whole body after the detoxification process in the liver. At the same time, the hepatobiliary system can secrete bile acids and other substances into the intestine to participate in the regulation of hormone levels and immune response, and affect intestinal homeostasis. Then, the gut microbiome can convert the primary bile acids that are excreted into the intestine from the hepatobiliary system into secondary bile acids. Moreover, 95% of the bile acids can be reabsorbed by the intestinal wall, and then enter the hepatobiliary system again through the portal vein (Tripathi et al., 2018; Jiang et al., 2019; Chopyk and Grakoui, 2020). The liver defends against intestinal toxins such as lipopolysaccharides (LPS) and products of the gut microbiome by its innate immune system. Moreover, the liver regulates metabolism and immune response and affects intestinal function through bile secretion and enterohepatic circulation. Dysbiosis of the gut microbiome and increased permeability of the intestinal wall are closely related to hepatobiliary disease through immune responses. Similarly, hepatobiliary insufficiency is also positively correlated with dysbiosis of the gut microbiome (Figure 1).

### Human Microbial Dysbiosis in the Precancerous Diseases of Cholangiocarcinoma

It is well known that patients with bile duct stones, PSC, liver fluke infection, and bile duct obstruction have a higher risk of CCA tumorigenesis. Patients with these precancerous diseases of CCA have human microbial dysbiosis. In turn, human microbial dysbiosis will promote the progression of precancerous diseases to CCA (Clements et al., 2020).

#### Human Microbial Dysbiosis in Gallstones

Studies have shown that abnormal secretion and metabolism of cholesterol and bile acids are important reasons for the formation of gallstones (Wang et al., 2009). The gut microbiome can regulate the metabolism of bile acids to influence the formation of gallstones to vary degrees. On the contrary, bile duct stones can cause alterations in the secretion of bile and inflammation response, resulting in dysbiosis of the gut microbiome (Sayin et al., 2013).

Keren et al. (2015) analyzed the difference between patients with gallstones and healthy controls in the gut microbiome. They found that the diversity of gut microbiome was significantly decreased in patients with gallstones. In addition, *Oscillospira* was enriched, while *Roseburia* was decreased when compared with healthy controls. Then, the increased level of bile acids in feces was detected; at the same time, the abundance of *Oscillospira* was negative with primary bile acids and correlated positively with secondary bile acids. In contrast, the correlation between *Bacteroides* and bile acids displayed the opposite trend to *Oscillospira* (Table 1). In another study, feces, bile, and gallstones from patients were collected and induced to 16S gene sequences. Proteobacteria was significantly increased in the gut microbiome compared with the healthy controls, while *Faecalibacterium*, *Lachnospira*, and *Roseburia* were decreased. Meanwhile, they reported that the bacteria diversity of the gut microbiome was significantly lower than that of the bile (Wu et al., 2013). The presence of dysbiosis in gut microbiome may be a key factor in the formation of gallstones. A large number of bacteria also exist in the bile and gallstones (Hazrah et al., 2004; Peng et al., 2015; Molinero et al., 2019). In addition, there was a difference in the bile microbiome between different types of gallstones (Abeysuriya et al., 2008). It was reported that the species of bile microbiome were largely consistent with that of the gut microbiome at the phylum level (Wu et al., 2013). There was no difference in the α and β diversity of the bile microbiome between patients with gallstones and healthy controls (Kim et al., 2021). Then, *Pseudomonas*, *Bacillus*, *Enterococcus*, and *Acinetobacter* were found as the dominant genera in the bile (Peng et al., 2015). Moreover, the level of *Enterococcus* was significantly higher in patients with gallstones than that in other causes of biliary obstruction (Kim et al., 2021).

**TABLE 1:** Dysbiosis in the human microbiome of cholangiocarcinoma and its precancerous diseases.

**Hepatobiliary disease**	**Type of the human microbiome**	**Dysbiosis in the microbiome**	**Clinical significance**	**Reference**
Gallstones	Gut	*Oscillospira* ↑ *Roseburia* ↓ *Proteobacteria* ↑ *Faecalibacterium* ↓ *Lachnospira* ↓ *Roseburia* ↓	Human microbial dysbiosis happened in patients with different types of gallstones	Wu et al., 2013; Keren et al., 2015
	Bile	*Pseudomonas* ↑ *Bacillus* ↑ *Enterococcus* ↑ *Acinetobacter* ↑		Peng et al., 2015; Kim et al., 2021
	Gallstones	*E. coli* ↑ *Pseudomonas* ↑		Lee et al., 1999
Primary sclerosing cholangitis	Gut	*Veillonella* ↑ *Exophiala* ↑ *Saccharomyces cerevisiae* ↓ *Oscillospira* ↑ *Anaeroplasma* ↑ *Ruminococcus* ↑ *Akkermansia* ↑ *Allobaculum* ↓ *Mucispirillum* ↓ *Anaerostipesa* ↓ *Coprococcus* ↓	Human microbial dysbiosis may influence disease progression. The human microbiome served as a non-invasive diagnostic biomarker for PSC	Sabino et al., 2016; Kummen et al., 2017, 2021; Schrumpf et al., 2017; Lemoinne et al., 2020
	Bile	*Proteobacteria* ↑ *Enterococcus* ↑ *Staphylococcus* ↑ *Neisseria* ↑		Liwinski et al., 2020
Liver fluke infection	Bile	*Lactobacillus* spp. ↑ *Aggregatibacter spp*. ↑ *Klebsiella spp*. ↑ *Treponema spp*. ↑ *Staphylococcus equorum* ↑ *Treponema socranskii* ↓ *Streptomyces sp*. ↓ *Xanthobacter sp*. ↓ *Flectobacillus sp* ↓	Liver fluke infection was associated with human microbial dysbiosis and promoted diseases progression	Saltykova et al., 2016
	Bile duct tissues	*Bifidobacteriaceae* ↑ *Enterobacteriaceae* ↑ *Enterococcaceae* ↑		Chng et al., 2016
Choledochal cysts	Bile	*Escherichia coli ↑ Klebsiella species ↑*	Choledochal cysts contributed to the development of CCA by inducing human microbial dysbiosis	Kaneko et al., 2005
Cholangiocarcinoma	Gut	*Alloscardovia* ↑ *Peptostreptococcaceae* ↑ *Actinomyces* ↑ *Lactobacillus* ↑ *Ruminococcus* ↓ *Leuconostocaceae*↓	The increased lipopolysaccharide-producing genera and cholestasis promoted tumor progression. The human microbiome served as a non-invasive diagnostic biomarker for CCA	Jia et al., 2019
	Bile	*Nitrospirae* ↑ *Gemmatimonadetes* ↑ *Geobacillus* ↑ *Bacteroides* ↑ *Enterococcus faecalis* ↑ *Enterococcus faecium* ↑ *Enterobacter cloacae* ↑ *Escherichia coli* ↑		Chen et al., 2019; Saab et al., 2021
	Bile duct tissues	*Dietziaceae* ↑ *Pseudomonadaceae* ↑ *Oxalobacteraceae* ↑ *Bifidobacteriaceae* ↑ *Enterobacteriaceae* ↑ *Enterococcaceae* ↑		Chng et al., 2016
	Blood	*Cyanobacteria* ↑ *Bacteroidetes* ↑ *Actinobacteria* ↑ *Firmicutes* ↑ Proteobacteria ↑		Lee et al., 2020

Surprisingly, the microbiome in the gallstones has been detected. Many species of *E. coli* and *Pseudomonas* were found in the cholesterol gallstones (Lee et al., 1999). Alterations in the microbiome of gallstones in patients with cholesterol gallstones were identified over time (Swidsinski et al., 1998). These findings help to figure out the development mechanism of cholesterol stones. In sum, the human microbial dysbiosis was involved in the process of the occurrence, progression, and deterioration of gallstones.

#### Human Microbial Dysbiosis in Primary Sclerosing Cholangitis

Primary sclerosing cholangitis is a chronic cholestatic liver disease. Approximately 70% of patients also suffer from inflammatory bowel disease. In addition, the inflammation of the colon causes the loss of the intestinal mucosal barrier, leading intestinal bacteria to enter the biliary tract *via* the portal vein, which contributes to infection of the biliary tract and progression of PSC. Studies have shown that the risk of CCA in patients with PSC is hundreds of times that of patients without PSC (Dyson et al., 2018).

A total of 543 stool samples, which included patients with PSC and healthy controls, were collected and induced to the 16S rRNA gene sequencing by Kummen et al. (2017). They identified the characteristics of the gut microbiome in patients with PSC. The diversity of gut microbiome in patients with PSC was significantly reduced compared with healthy controls, but there was no difference in the diversity of gut microbiome of in patients with PSC and with and without IBD. Comparing the relative abundance of the gut microbiome at the genus level, the data showed that *Veillonella* increased significantly in PSC (Kummen et al., 2017). And the abundance of the *Veillonella* genus was positively correlated with the Mayo risk score, which indicates the severity of the disease. Interestingly, an operational taxonomic unit (OUT), which belonged to the *Enterococcus* genus, was positively related to the serum level of alkaline phosphatase, which was regarded as a biomarker of disease severity (Sabino et al., 2016). Metagenomic shotgun sequencing showed that the bacteria genes that regulated the synthesis of vitamin B6 and branched-chain amino acids were significantly decreased in patients with PSC, and this was validated in the serum metabolomics (Kummen et al., 2021). Notably, in addition to bacteria, fungi were also involved in the dysbiosis of the gut microbiome in patients with PSC. Lemoinne et al. (2020) identified a relative increase in the diversity of fungal species. They found that the relative abundance of *Exophiala* increased significantly, and that of *Saccharomyces cerevisiae* decreased significantly (Lemoinne et al., 2020). Schrumpf et al. (2017) constructed the mouse model of spontaneous bile duct inflammation and reported that at the genus level, *Oscillospira*, *Anaeroplasma*, *Ruminococcus*, and *Akkermansia* were significantly enriched compared with control mice. In contrast, *Allobaculum*, *Mucispirillum*, *Anaerostipes*, and *Coprococcus* were decreased (Schrumpf et al., 2017).

Meanwhile, microbial dysbiosis also existed in the bile of patients with PSC. Bile samples were collected using endoscopic retrograde cholangiography from patients with PSC and controls. Analysis results showed a decrease in diversity of the species of bile microbiome in patients with PSC vs. controls. And an enrichment of *Enterococcus faecalis* was found in patients with PSC. Moreover, the relative abundance of *E. faecalis* was positively related to the level of the harmful taurolithocholic acid (Liwinski et al., 2020). Dysbiosis in the biliary was associated with increased concentrations of the proinflammatory and potential carcinogen taurocholic acid. This may provide new insights for the treatment of PSC. The above research indicated that there was a close relationship between human microbial dysbiosis and the occurrence, progression, and deterioration of PSC.

#### Human Microbial Dysbiosis in Liver Fluke Infection

Liver fluke infection, which is defined as the group 1 carcinogen, will greatly increase the risk of CCA. Liver flukes are located in the biliary tree after entering the human body. And chronic infection of liver flukes will lead to tumorigenesis. In addition, chronic infection causes alterations in the human microbiome (Saltykova et al., 2018).

Saltykova et al. (2016) collected 56 bile samples from gallstone patients with or without liver fluke infection and conducted the 16S rRNA sequence. They claimed that *Lactobacillus* spp., *Aggregatibacter* spp., *Klebsiella* spp., *Treponema* spp., and *Staphylococcus equorum* were enriched in the bile from infected patients compared with non-infected patients, whereas *Treponema socranskii*, *Streptomyces* sp., *Xanthobacter* sp., and *Flectobacillus* sp. were increased in the uninfected group (Saltykova et al., 2016). Another study showed that the Shannon diversity index was significantly increased in the normal bile duct tissues because of the infection of liver fluke (Chng et al., 2016). Surprisingly, they found that Bifidobacteriaceae, Enterobacteriaceae, and Enterococcaceae were enriched in the liver fluke-associated bile duct tissues. These indicated that the Bifidobacteriaceae in the bile duct tissue may be introduced by liver flukes. In addition, the increased potential for producing bile acids and ammonia was associated with the alterations in the microbiome. In conclusion, the human microbiome was related to the process of the occurrence, progression, and deterioration of liver fluke infection.

#### Human Microbial Dysbiosis in Choledochal Cysts

Choledochal cysts (CCs) are cystic dilatation of the biliary tract which often occurs in children. CC is also known as a rare precancerous lesion of CCA (Soares et al., 2015). Kaneko et al. (2005) collected 122 bile specimens from patients with CCs and carried out bacterial culture. The results showed that gram-negative enterobacteria, such as *Escherichia coli* and *Klebsiella* species, and non-enteric bacteria were enriched in the bile. Chronic bile infection is important for stone formation and tumorigenesis. The detailed mechanism needs further research.

#### Human Microbial Dysbiosis in Cholangiocarcinoma

The human microbiome, including gut, bile, bile duct tissue, and blood microbiome, was closely related to the tumorigenesis and progress of CCA. Jia et al. (2019) recruited 84 volunteers, including 28 patients with iCCA, 28 patients with hepatocellular carcinoma (HCC), 16 patients with liver cirrhosis, and 12 healthy controls. They found that Firmicutes, Bacteroidetes, Actinobacteria, and Verrucomicrobia were the most dominant gut microbiome at the phylum level. Additionally, the gut microbiome of the patients with ICC had the highest diversity (both α-diversity and β-diversity). Compared with other groups, at the genus level, *Alloscardovia*, Peptostreptococcaceae, *Actinomyces*, and *Lactobacillus* were significantly enriched in the patients with ICC. Also, *Ruminococcus* and Leuconostocaceae were enriched in the healthy controls. Furthermore, they identified the association between specific bacterial characteristics and clinical characteristics. The genera *Lactobacillus* and *Alloscardovia* were positively correlated with tauroursodeoxycholic acid. Plasma tauroursodeoxycholic acid was negatively correlated with the genus *Pseudoramibacter* and with survival time but positively correlated with vascular invasion. There were positive correlations among plasma tricarboxylic acid, IL-4, and vascular invasion.

When it comes to the bile microbiome of CCA, the bile samples from patients with PCC and gallstones were collected and compared. The analysis results showed that *Pyramidobacter*, *Klebsiella*, *Bacteroides*, and *Enterococcus* were the most dominant bacteria in the bile of patients with PCC. Then, compared with patients with gallstones, the phylum Nitrospirae and Gemmatimonadetes and the genus *Geobacillus* and *Bacteroides* were enriched in patients with PCC (Chen et al., 2019; Saab et al., 2021). Meanwhile, Jan Bednarsch et al. (2021) studied the bile microbiome of patients with PCC. They identified a large number of bacterial colonization in the bile of patients with PCC using bacterial culture. Notably, *E. faecalis, Enterococcus faecium, Enterobacter cloacae*, and *E. coli* were the most common bacterial species.

The microbiomes of the bile duct tissue in patients with CCA were characterized using 16S rRNA sequencing. The result showed that Dietziaceae, Pseudomonadaceae, and Oxalobacteraceae were relatively abundant microbiomes in the CCA tissues compared with the other nearby tissues (Chng et al., 2016). In addition, the difference in the tissue microbiomes between liver fluke-associated CCA and non-liver fluke-associated CCA was identified. It was reported that Bifidobacteriaceae, Enterobacteriaceae, and Enterococcaceae were significantly enriched in the fluke-associated CCA tissues. Furthermore, surprisingly, the gene functional analysis found that the functions of producing bile acid and ammonia were significantly enhanced in CCA tissue microbiome (Chng et al., 2016).

Surprisingly, the blood microbiome of patients with CCA was also identified by Lee et al. (2020). At the phylum level, the most dominant bacteria were Cyanobacteria, Bacteroidetes, Actinobacteria, Firmicutes, and Proteobacteria in patients with CCA (Lee et al., 2020). Then, they constructed a diagnostic model using blood microbiome for patients with CCA. But the blood microbiome is poorly understood, and more studies are needed to understand alterations and mechanisms.

### The Effect of Helicobacter Pylori on Cholangiocarcinoma

*Helicobacter pylori*, which is a gram-negative bacteria, is closely related to peptic ulcer and gastric cancer. However, more researchers believed that *H. pylori* plays an important role in tumors other than gastric cancer (Pellicano et al., 2008; Pandey and Shukla, 2009; Suzuki et al., 2011). In CCA, studies showed that *H. pylori* was associated with the possible increase risk. The bile and bile duct tissue from 19 patients with hepatobiliary cancer and 19 controls were collected and induced to PCR (Fukuda et al., 2002). *H. pylori* was identified in 10 patients (52.6%) and 3 controls (15.7%). At the same time, the multiple regression analysis showed that there is a close correlation between the presence of *H. pylori* and the increase of the proliferating cell nuclear antigen marker index in the bile duct epithelium. Bulajic et al. (2002) have also reached a similar result that *H. pylori* in the bile is closely related to an increased risk of malignant biliary disease.

Additionally, *H. pylor*i in the blood of patients with CCA has also been detected. Murphy et al. (2014) collected the serum from 64 patients with biliary tract cancer, 122 patients with HCC, and 224 healthy controls and analyzed the seropositivity to *Helicobacter* species. Then, they reported that the prevalence of seropositivity in healthy controls was 88%, while the prevalence in ICC and PCC rose to 97% and 96%, respectively. And the odds ratios were 7.01 (95% CI: 0.79–62.33) and 10.67 (95% CI: 0.76–150.08), respectively. Their research proved that seropositivity of *H. pylori* protein had a close relationship with the increased risk of CCA.

### Human Microbial Dysbiosis Promotes the Progress of Cholangiocarcinoma and Its Precancerous Diseases

The gut microbiome is involved in the process of antitumor immunity in CCA. Microbiome dysbiosis was detected in the mouse model of CCA. Additionally, the intestinal barrier function was destroyed, allowing intestinal bacteria and LPSs to enter the liver through the portal vein. Then, the TLR4-dependent mechanism and the CXCL1-CXCR2 axis led to the accumulation of polymorphonuclear myeloid–derived suppressor cells (PMN-MDSC), thereby promoting the immune escape and progression of CCA. On the contrary, healthy mice orally took neomycin to remove part of the gut microbiome and change the structure. The results showed that the reduction in the expression of CXCL1 and suppression of the aggregation of PMN-MDSC resulted in inhibiting tumor growth (Zhang et al., 2021). Thus, gut microbial dysbiosis promotes the progress of CCA.

The bile microbiome played an important role in gallstone disease. There were differences in the pathogenesis of different gallstones. The biliary microbiome promoted the formation of brown pigment gallstones. Then, LPSs, oxygen free radicals, oxysterols, and prostaglandins were involved in the formation of gallstones. In addition, the appearance of cholesterol gallstones was closely related to *H. pylori* and *Salmonella*. Furthermore, the metabolites of bile microbiome have been identified in promoting the formation of gallstones. Thus, bile microbial dysbiosis promotes the progress of gallstones (Yoshida et al., 2003; Haigh et al., 2006; Peng et al., 2015; Wang et al., 2018).

Gut microbial dysbiosis promotes the progress of PSC. In a PSC mouse model, researchers found gut microbial dysbiosis in PSC mice. Additionally, the results showed dysfunction of intestinal barrier, increased bacterial translocation, and activation of NLRP3 inflammasome. To identify the detail mechanism, they transplanted the feces of PSC mice into healthy mice. Surprisingly, the recipient healthy mice showed obvious liver damage, highlighting that the gut microbial dysbiosis promotes the progress of PSC (Liao et al., 2019; Dean et al., 2020). Thus, dysbiosis of the human microflora promotes the progress of CCA and its cancerous diseases.

### Human Microbiome Serves as a Non-invasive Diagnostic Biomarker for Cholangiocarcinoma

The diagnostic value for the human microbiome has been reported in many diseases, such as type 2 diabetes (Qin et al., 2012), autoimmune hepatitis (Wei et al., 2020), liver cirrhosis (Qin et al., 2014), and colorectal cancer (Yu et al., 2017). A total of 486 fecal samples of patients with HCC and controls from Central, East, and Northwest China have been collected and induced to 16S rRNA sequencing (Ren et al., 2018). Then, the characteristics were described and the alterations of the gut microbiome in patients with HCC were figured out. Compared with cirrhosis, Actinobacteria phylum, *Gemmiger* genus, and *Parabacteroides* genus were significantly enriched in HCC. In addition, a diagnostic model was constructed using the random forest model based on the 30 optimal microbial biomarkers. A diagnostic efficiency of 80.64% was obtained between HCC and non-HCC samples in the discovery cohort. Moreover, high diagnostic efficiencies were also obtained in the validation cohort and independent diagnosis cohort. In another study, we illustrated the gut and oral microbial characteristics of patients with coronavirus disease 2019 (COVID-19) (Ren et al., 2021). In the meanwhile, the diagnostic value of human microbiomes in COVID-19 was identified and validated. Jia et al. (2019) constructed a microbial non-invasive diagnostic model for CCA by the logistic regression model. They selected two key genera (*Lactobacillus* and *Alloscardovia*) to construct the diagnostic model, and the ACU value was achieved at 0.968, 0.965, and 0.987 to distinguish CCA from HCC, cirrhosis, and healthy controls, respectively (Jia et al., 2019). The diagnostic value of other human microbiome except the gut microbiome in CCA needs further exploration.

### The Prospect of Translational Medical Research of Human Microbiome of Cholangiocarcinoma

In recent years, human microbiome and high-throughput sequencing have contributed much to the diagnosis, prognosis, and treatment of CCA (Yu and Schwabe, 2017; Tripathi et al., 2018). But there are many unknown facts about the interaction between the human microbiome and CCA. With the progress of research in microbiome diversity, studies in future should concentrate on the functions of microbiome and their metabolites, so as to further analyze the mechanism of between microbiome and diseases. At the same time, the above results need to be verified in a large number of rigorous animal experiments. Sterile animals would be a good choice, which can eliminate the influence of the original gut microbiome and the external environment. Moreover, in sterile animals, fecal microbiota transplantation is used to validate the causal relationship between microbiome and CCA.

The microbiome can serve as a potential treatment for CCA in future. We can use drugs that will interfere with the structure of the microbiome and microbiota transplantation to improve the human microbiome dysbiosis (Bajaj and Hays, 2019; Nicoletti et al., 2020; Shah et al., 2020). With the deepening of the understanding of the link between the gut microbiome and the liver, it will provide new opportunities for the development of new CCA treatment strategies.

## Author Contributions

ZY and ZR designed the study. BR, TR, and XW provided equal contributions to the data curation and writing of the manuscript. HW, YZ, YS, SL, ZR, and ZY revised the manuscript. All authors contributed and approved the submitted version of the manuscript.

## Conflict of Interest

The authors declare that the research was conducted in the absence of any commercial or financial relationships that could be construed as a potential conflict of interest.

## Publisher’s Note

All claims expressed in this article are solely those of the authors and do not necessarily represent those of their affiliated organizations, or those of the publisher, the editors and the reviewers. Any product that may be evaluated in this article, or claim that may be made by its manufacturer, is not guaranteed or endorsed by the publisher.
